# Dental Advertisements

**Published:** 1848-10

**Authors:** 


### DENTAL ADVERTISEMENTS.

We have received from a friend in Georgia, quite a bundle of Dental advertisements, cards, placards, handbills, &c., &c. We had supposed that the good taste (if not of those claiming to be members of the profession) at least of the public, had driven more generally such displays of dental buffoonery out of practice. We measured one of the bills, and it is only twelve by eighteen inches, having attached thereto as reference, the names of seven doctors, three reverends, two honorables, two colonels, two esquires, and one general. This is admirably contrived, for one general is enough to an army. We have no doubt but that *Sparta* fell under this attack. Just think of the horrid implements of warfare, turnkeys, forceps, and full upper sets of teeth, first slay, and then eat.

We have next a more sizable sheet, eight by ten, evidently from the hands of a young beginner, just from writing school, tickled half to death to see his name in print, introduced with the flourishing title of Doctor. In filling up his handbill his trepidation has been such that his writing is scarcely legible. He gives us a fine dissertation on extracting, cleansing, filing, and plugging of teeth—then strikes off into the causes of disease. Here we will give a small specimen of his erudition, "Tartar is one of the most frequent *causes* of *diseases* in the Teeth; it acts directly (chemically) in the destruction of them, and is one of the most powerful *causes of diseases* in the gums, periosteum, and alveoli." Here our learned doctor has found out a new principle in chemistry, by which the phosphate of lime in the tartar destroys the phosphate of lime in the tooth. We think the discovery worthy of a medal. We have net something so modest that we cannot omit it here, from the card of Dr. F., "all operations performed on the most modern *improved scientific* principles, with the least possible pain, *and correct professional skill.*" We suppose he here means the "least possible correct professional skill," as well as "pain." We admire honesty as well as modesty.

We had in our city a year or two since a flaming Doctor, Dentist, &c., who, we supposed at the time, had cut out for himself a new theory to sustain his practice, but here we have the saying that "there is nothing new under the sun," forcibly illustrated. Many of our readers at least in this city, will recognize the following extract, which we make from one of the old advertisements sent us:—

"*Extracting Teeth.*—The practice adopted by many Dentists and Medical Practitioners, of extracting all painful and decayed teeth, Dr. P— in justice to humanity, and dental science, unqualifiedly condemns. It is an unfeeling practice, evincing an ignorance of well established principles in the treatment of complicated caries; a practice that outrages common sense—is at war with physiology, and often attended with consequences of an appalling character. A very *large majority* of the molar or jaw teeth daily extracted, would be speedily restored to ease and usefulness, under an enlightened and judicious treatment, and rendered valuable for many years, and perhaps for life. *This fact cannot be urged too strongly upon the consideration of females, who suffer so much from tooth ache,* and who justly contemplate the extraction of a tooth with fear and trembling."

It is remarkable that the system of physiology adopted by Dr. H. recently of this city, and Dr. P. of the south, should lead both to the same identical language in the publication of their views, when several years have intervened.

Quackery can scarce find a new dress any more, to appear in. We have not the time at present, to go further into these documents.—We, however, see a great many new improvements and discoveries announced, claiming all and a little more. We hope that dental science will soon be illuminated by the publication to the world, of some of these many newly discovered, but now locked up principles, which are ultimately to revolutionize dental practice. We may analyze, at another time, some more of these productions. Will our friend accept our thanks for these favors, and send us soon something substantial for the Register. We hope the present number will meet his views in typography, and every other respect.—Cin. Ed.

